# Thermal Stability of Hole-Selective Tungsten Oxide: *In Situ* Transmission Electron Microscopy Study

**DOI:** 10.1038/s41598-018-31053-w

**Published:** 2018-08-23

**Authors:** Haider Ali, Supriya Koul, Geoffrey Gregory, James Bullock, Ali Javey, Akihiro Kushima, Kristopher O. Davis

**Affiliations:** 1 Department of Materials Science and Engineering, University of Central Florida, Orlando, FL USA; 2 Florida Solar Energy Center, University of Central Florida, Cocoa, FL USA; 3 Department of Electrical Engineering and Computer Science, University of California, Berkeley, CA USA

## Abstract

In this study, the thermal stability of a contact structure featuring hole-selective tungsten oxide (WO_x_) and aluminum deposited onto *p*-type crystalline silicon (c-Si/WO_x_/Al) was investigated using a combination of transmission line measurements (TLM) and *in situ* transmission electron microscopy (TEM) studies. The TEM images provide insight into why the charge carrier transport and recombination characteristics change as a function of temperature, particularly as the samples are annealed at temperatures above 500 °C. In the as-deposited state, a ≈ 2 nm silicon oxide (SiO_x_) interlayer forms at the c-Si/WO_x_ interface and a ≈ 2–3 nm aluminum oxide (AlO_x_) interlayer at the WO_x_/Al interface. When annealing above 500 °C, Al diffusion begins, and above 600 °C complete intermixing of the SiO_x_, WO_x_, AlO_x_ and Al layers occurs. This results in a large drop in the contact resistivity, but is the likely reason surface recombination increases at these high temperatures, since a c-Si/Al contact is basically being formed. This work provides some fundamental insight that can help in the development of WO_x_ films as hole-selective rear contacts for *p*-type solar cells. Furthermore, this study demonstrates that *in situ* TEM can provide valuable information about thermal stability of transition metal oxides functioning as carrier-selective contacts in silicon solar cells.

## Introduction

To achieve high conversion efficiencies (>25%) for a single junction crystalline silicon (c-Si) solar cell, it is essential to couple low recombination velocities in the bulk and at the Si surface with suitable carrier selectivity at both electron and hole contact regions. These objectives can be achieved by inserting carrier selective contacts at both the front and rear surfaces of a silicon heterojunction cell. They help in passivating the Si surface by reducing surface recombination and they are carrier-selective in nature, meaning that they allow only one type of carrier, i.e., either of electron or holes to pass through it while blocking the other^1–6^.

Traditionally, carrier selective contacts have been realized using doped hydrogenated amorphous silicon (a-Si:H), wherein a-Si:H(*n*) and a-Si:H(*p*) act as electron-selective and hole-selective contacts respectively. These contacts are typically used in combination with a thin a-Si:H(*i*) or SiO_2_ passivation layer. However, such cells suffer from certain inherent drawbacks associated with a-Si:H such as thermal instability, parasitic photon absorption, complicated deposition processes and high fabrication costs^1,7–11^.

Transition metal oxides have emerged as promising alternatives to doped a-Si:H for use as carrier selective contacts in silicon heterojunction cells. Depending on the valence-band and conduction-band offset between the metal oxide and Si, transition metal oxides can be employed as either an electron-selective or a hole-selective contact. For instance, titanium oxide (TiO_2_) has a small conduction band offset (Δ*E*_c_ = 0.05 eV), which provides a low barrier for electrons to pass through the TiO_2_ layer and a large valence-band offset (Δ*E*_v_ = 2.0 eV) that results in holes being blocked. Due to this reason, TiO_2_ (<5 nm) has been employed as an electron-selective rear contact for an *n*-type cell with very promising results^1,7,8,12,13^.

On the other hand, if metal oxides have a wide band gap and high work function, the strong work function difference between Si and metal oxide induces a strong upward band bending in the c-Si which results in electrons being blocked. Because of this reason, sub-stochiometric metal oxides such as molybdenum oxide (MoO_x_) and tungsten oxide (WO_x_) have emerged as promising candidates for use as hole-selective contacts. Because of their wide band gap (>3 eV) and consequently their high transparency, these materials have been employed as hole-selective front contacts in combination with a transparent conducting oxide (TCO) layer such as hydrogenated indium oxide (IO:H) or indium tin oxide (ITO)^2,14–18^. However, a major limitation of these contact structures is that they are sensitive to low temperature annealing resulting in degradation of device performance. Although, the origin of these losses is yet to be fully understood, it is likely due to a reduction in the work function driven by hydrogenation and/or a lower oxygen concentration resulting in non-ideal hole-selectivity^16,19^.

Although, wide band gap and high work function transition metal oxides s (e.g., MoO_x_, WO_x_) in combination with TCO (e.g., IO:H, ITO) have been widely investigated as hole-selective front contacts for *n*-type solar cells; their application as potential hole-selective rear contacts are yet to be fully explored. Recently, Lee *et al*. demonstrated that WO_x_/Al can be a potential candidate to be employed as a full area hole-selective rear contact for a *p*-type Si solar cells^20^. In the present work, the thermal stability of a c-Si/WO_x_/Al contact structure was investigated while being subjected to annealing temperatures up to 700 °C with the help of *in situ* transmission electron microscopy (TEM). The contact resistivity at various annealing temperatures was measured by transmission line measurements (TLM). The objective is to achieve an in-depth understanding of the mechanisms influencing the stability of WO_x_/Al contacts when subjected to high temperature annealing at the microscopic scale.

**Figure 1:**
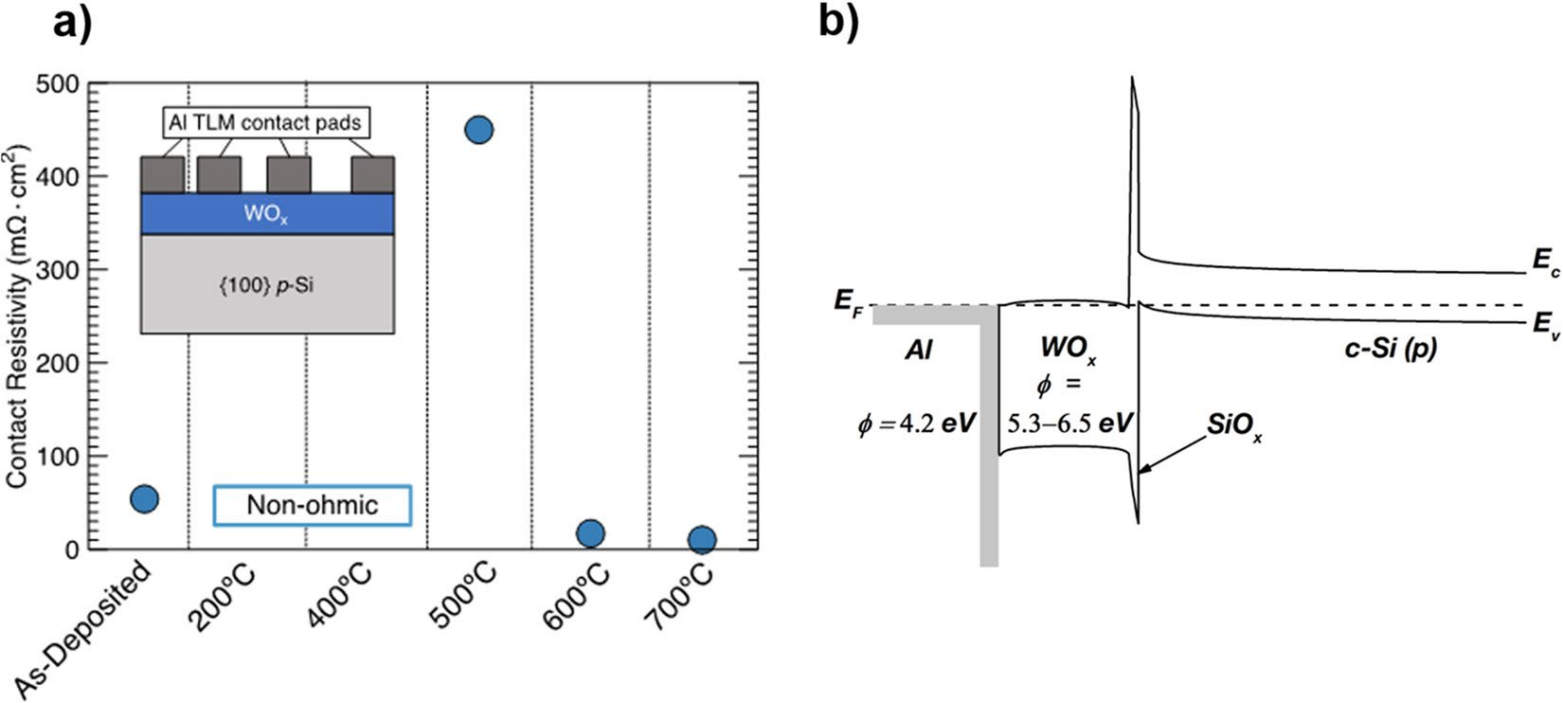
(**a**) Contact resistivity at various annealing temperatures obtained by TLM and (**b**) the energy band diagram of the WO_x_/Al contact to *p-*type c-Si.

**Figure 2:**
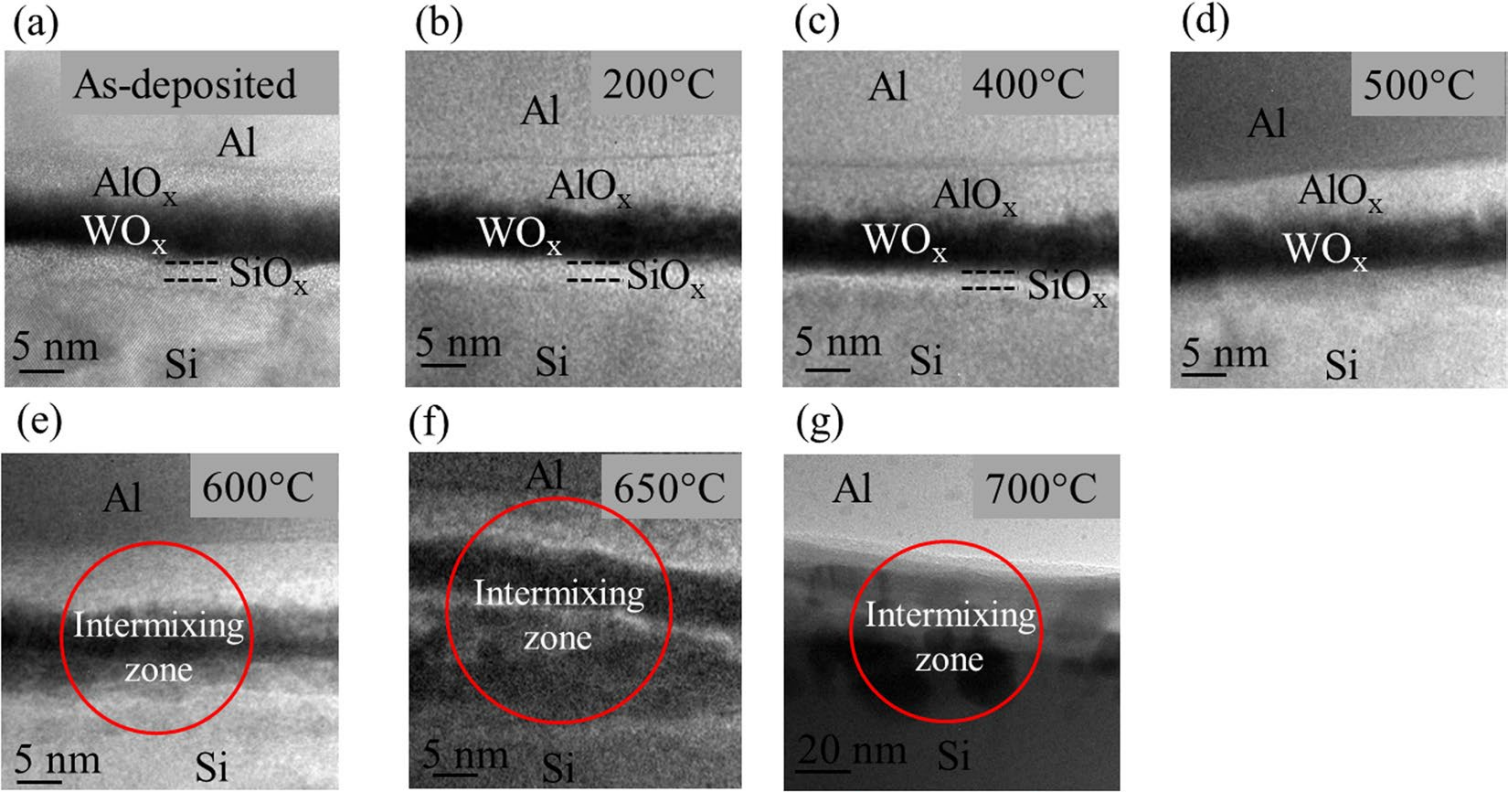
Cross-sectional HRTEM micrograph of c-Si/WO_x_/Al structure obtained by *in situ* TEM studies (**a**) as-deposited, (**b**) 200 °C, (**c**) 400 °C, (**d**) 500 °C, (**e**) 600 °C, (**f**) 650 °C, (**g**) 700 °C.

The values of contact resistivity measured at various annealing temperatures are shown in Fig. 1(a) and the inset illustrates the schematic of TLM structures employed for contact resistivity measurements. A typical energy band diagram of WO_x_/Al contact to p-type c-Si is shown in Fig. 1(b)^21^. In the as-deposited state, the electrical behavior of the contact is ohmic and a contact resistivity value of 54 mΩ·cm^2^ was obtained. Moreover, it is evident from cross-sectional HRTEM micrographs shown in Fig. 2(a) that even in the as-deposited state, an AlO_x_ interlayer is formed at the WO_x_/Al interface. A similar behavior has been previously reported for Al/TiO_2_ contacts as well^1,8^. Likewise, in the case of WO_x_/Al, because of the higher oxygen affinity of Al as compared to the oxygen affinity of W, diffusion of oxygen is more likely to occur from WO_x_ towards Al resulting in formation of AlO_x_ interlayer. This can lead to the creation of defect states such as oxygen vacancies within the WO_x_ layer. Moreover, a ≈ 2 nm SiO_x_ interlayer is also observed at the c-Si/WO_x_ interface. This is consistent with observation made by Sachetto *et al*. It is obvious that the formation of a SiO_x_ interlayer occurs during deposition of WO_x_ by thermal evaporation and can be explained by thermodynamic considerations^22^. An effective carrier lifetime (*τ*_eff_) value of 53 µs at an injection level (∆*n*) of 1·10^15^ cm^−3^ has been reported for as-deposited WO_x_/Al^20^. It can be concluded that, in the as-deposited state, although a SiO_x_ interlayer forms, this layer provides very little surface passivation, and likely has a reasonably high interface defect density. Additionally, both the work function and film conductivity are sufficiently high to form an ohmic contact with a reasonably low contact resistivity in the as-deposited state.

When the sample is annealed to 200 °C no significant change is observed (Fig. 2(b) and video 2(b)). However, TLM results show that contact structure becomes non-ohmic. One possible explanation for the non-ohmic transport is a reduction in the work function upon annealing in ambient air resulting in the formation of a barrier to hole collection. Additionally, if oxygen is diffusing from WO_x_ to the SiO_x_ interlayer, the SiO_x_ interlayer may become more insulating^22^. This, however, requires further investigation. With respect to surface passivation of the c-Si/WO_x_/Al contact stack, it has been previously reported that *τ*_eff_ drops from 53 µs to 16 µs (∆*n* = 1·10^15^ cm^−3^) when annealed at 200 °C^20^.

When the annealing temperature is increased to 400 °C, no apparent structural change is observed in the contact stack and the electrical behavior remains non-ohmic (Fig. 2(c) and video 2(c)). However, previous work on c-Si/WO_x_/Al reported that *τ*_eff_ actually increases upon annealing at 400 °C based on photoluminescence images of asymmetrical test structure, although it is unclear whether this increase was due to improved passivation of the SiO_x_ interlayer at the WO_x_ (i.e., rear) side of the device, or due to an improvement in the surface passivation at the front side of the device^20^. Yang *et al*. have reported that in case of SiO_2_/TiO_2_/Al rear contacts, annealing at 350 °C is essential to activate the surface passivation of SiO_2_^7,8^. Therefore, it is possible that when the annealing temperature is increased to 400 °C, it activates the passivation mechanism of SiO_x_ which results in increase in *τ*_eff_.

When the annealing temperature is further increased to 500 °C, interfaces becomes diffused, indicating that significant phase changes may begin to occur at 500 °C (Fig. 2(d) and video 2(d)). Furthermore, the TLM studies revealed that the behavior of the contact structure changes from non-ohmic to ohmic, although the contact resistivity value obtained was significantly higher at 450 mΩ·cm^2^. It has been previously reported that crystallization of WO_x_ occurs at 400 °C^20^, although no evidence of crystallization was found during *in situ* TEM observation. It is likely that WO_x_ begins to crystallize at 400 °C and continues when further annealed to 500 °C. However, short annealing times (≈10 minutes) are insufficient for the WO_x_ to undergo complete crystallization, even at 500 °C^20^. Therefore, ohmic behavior upon annealing to 500 °C with a relatively higher contact resistivity can be attributed to partial crystallization of WO_x_.

As already discussed, when annealing temperature is increased beyond 500 °C, it is likely that the phase changes begins to occur in WO_x_/Al. Furthermore, at 600 °C, intermixing takes place between SiO_x_, WO_x_, AlO_x_ and Al layers (Fig. 2(e) and video 2(e)). At 650 °C, the layers become indistinguishable (Fig. 2(f) and video 2(f)). At 700 °C, intermixing between Si, WO_x_ and Al appears to be near completion and Al is in direct contact with Si (Fig. 2(g)). This can be explained in terms of thermodynamic considerations. It is well known that the melting point of Al is 660 °C, and in the range of 100–150 °C below the solvus line, Al begins to lose its integrity and diffusion begins. This explains why diffused interfaces were observed at a temperature of 500 °C. Al is the faster diffusing species than W because of the much smaller atomic radius. And as the annealing temperature is increased beyond 500 °C, Al atoms diffuse towards Si substrate with an exponentially increasing rate of diffusion with temperature. Eventually, at 650 °C, which is near the melting point of pure Al, significant intermixing occurs. Finally, at 700 °C, the intermixing process nears completion and the Al layer comes in direct contact with the Si. Therefore, much lower contact resistivity values of 17 mΩ·cm^2^ and 10 mΩ·cm^2^ were obtained at 600 °C and 700 °C, respectively. This implies that drastic drop in contact resistivity upon annealing at 600 °C and beyond is most likely due to direct contact between Al and Si. However, this is an adverse effect on carrier selectivity and surface passivation, which is consistent with lower *τ*_eff_ values reported at 600 °C^20^, and basically negates the whole purpose of using WO_x_.

Overall, it emerged that although WO_x_/Al is a potential candidate to be employed as a hole-selective rear contact on a *p*-type cell, certain issues remain. To activate the passivation mechanism of SiO_x_, annealing samples at 400 °C may be sufficient, but WO_x_/Al becomes non-ohmic at that temperature. This can possibly be overcome by annealing WO_x_/Al for longer times to allow sufficient time for crystallization of WO_x_, which can make it more conductive, but care must be taken to not reach the point of intermixing resulting in essentially a c-Si/Al contact.

In summary, some fundamental insight that can help in the development of WO_x_ films as hole-selective rear contacts for p-type solar cells has been provided in this study. Furthermore, this work has successfully demonstrated that *in situ* TEM can provide valuable information about thermal stability of transition metal oxides employed as carrier-selective contacts in silicon solar cells.

## Experimental Section

WO_x_ thin films (<10 nm) were deposited on 5–10 Ω.cm p-type FZ {100} c-Si wafers under vacuum by thermal evaporation using a powder WO_3_ source. During the evaporation process, heating of the c-Si substrate was minimized and remained close to ambient temperature. This was followed by evaporation of 500 nm of Al to form the metal contact. For the contact resistivity measurements, TLM structures were fabricated, previously shown as an inset in Fig. 1(a). Dark I-V measurements were taken for the TLM contact pairs at different spacings using a Keithley 2400 Sourcemeter. This was done on samples exposed to different post-deposition annealing temperatures. This data was then used to extract the contact resistivity of the WO_x_ contact stack, and the data was corrected for current spreading due to the absence of an emitter using the extended TLM^23^.

For TEM studies, cross-sectional TEM specimens were prepared by the focused ion beam (FIB) milling technique using FEI 200 TEM FIB. Specimen lift-out was done *in situ* and attached to Cu grid. To monitor morphological changes occurring at elevated temperatures, the sample was analyzed using TEM and was heated *in situ*. The *in situ* TEM experiments was performed using a Gatan heating holder (Model 652) in combination with a FEI Tecnai F30 under operating voltage of 300 kV. The samples were annealed *in situ* up to a temperature of 700 °C with a heating rate of 50 °C/min and an annealing time for each temperature of 10 min. However, a major limitation of using a Gatan heating holder (652) is that it is not compatible with energy dispersive x-ray spectroscopy (EDX) systems. This is because the TEM specimen is surrounded by a furnace without any direct line of sight from the sample to the EDX detector due to which it is unable to detect characteristics x-rays essential for determination of chemical composition.

## Electronic supplementary material

Video 2(b)

Video 2(c)

Video 2(d)

Video 2(e)

Video 2(f)

## References

[1] Ali H, Yang X, Weber K, Schoenfeld WV, Davis KO (2017). Transmission Electron Microscopy Studies of Electron-Selective Titanium Oxide Contacts in Silicon Solar Cells. Microscopy and Microanalysis.

[2] Bivour M, Temmler J, Steinkemper H, Hermle M (2015). Molybdenum and tungsten oxide: High work function wide band gap contact materials for hole selective contacts of silicon solar cells. Solar Energy Materials and Solar Cells.

[3] Feldmann F (2014). Carrier-selective contacts for Si solar cells. Applied Physics Letters.

[4] Aberle AG (2000). Surface Passivation of Crystalline Silicon Solar Cells: A Review. Progress in Photovoltaics: Research and Applications.

[5] Ali, H., Moldovan, A., Mack, S., Schoenfeld, W. V. & Davis, K. O. Transmission electron microscopy based interface analysis of the origin of the variation in surface recombination of silicon for different surface preparation methods and passivation materials. *physica status solidi (a)*, 1700286 (2017).

[6] Ali H (2017). Influence of surface preparation and cleaning on the passivation of boron diffused silicon surfaces for high efficiency photovoltaics. Thin Solid Films.

[7] Yang X, Zheng P, Bi Q, Weber K (2016). Silicon heterojunction solar cells with electron selective TiO_x_ contact. Solar Energy Materials and Solar Cells.

[8] Yang X (2016). High-Performance TiO_2_-Based Electron-Selective Contacts for Crystalline Silicon Solar Cells. Advanced Materials.

[9] Bullock J (2014). Amorphous silicon passivated contacts for diffused junction silicon solar cells. Journal of Applied Physics.

[10] Bivour M, Reichel C, Hermle M, Glunz SW (2012). Improving the a-Si:H(p) rear emitter contact of n-type silicon solar cells. Solar Energy Materials and Solar Cells.

[11] Bullock J (2015). Simple silicon solar cells featuring an a-Si:H enhanced rear MIS contact. Solar Energy Materials and Solar Cells.

[12] Yang, X. & Weber, K. *In Photovoltaic Specialist Conference (PVSC), IEEE 42nd*. 1–4. (2015).

[13] Allen TG (2017). A Low Resistance Calcium/Reduced Titania Passivated Contact for High Efficiency Crystalline Silicon Solar Cells. Advanced Energy Materials.

[14] Gerling LG (2016). Transition metal oxides as hole-selective contacts in silicon heterojunctions solar cells. Solar Energy Materials and Solar Cells.

[15] Bullock J, Cuevas A, Allen T, Battaglia C (2014). Molybdenum oxide MoO_x_: A versatile hole contact for silicon solar cells. Applied Physics Letters.

[16] Geissbühler J (2015). 22.5% efficient silicon heterojunction solar cell with molybdenum oxide hole collector. Applied Physics Letters.

[17] Battaglia C (2014). Silicon heterojunction solar cell with passivated hole selective MoO_x_ contact. Applied Physics Letters.

[18] Gerling LG, Voz C, Alcubilla R, Puigdollers J (2016). Origin of passivation in hole-selective transition metal oxides for crystalline silicon heterojunction solar cells. Journal of Materials Research.

[19] Neusel L, Bivour M, Hermle M (2017). Selectivity issues of MoO_x_ based hole contacts. Energy Procedia.

[20] Lee C-Y, Aziz MIA, Wenham S, Hoex B (2017). Characterisation of thermal annealed W_O_x on p-type silicon for hole-selective contacts. Japanese Journal of Applied Physics.

[21] Mews M, Korte L, Rech B (2016). Oxygen vacancies in tungsten oxide and their influence on tungsten oxide/silicon heterojunction solar cells. Solar Energy Materials and Solar Cells.

[22] Sacchetto D (2017). ITO/MoO_x_/a-Si:H(i) Hole-Selective Contacts for Silicon Heterojunction Solar Cells: Degradation Mechanisms and Cell Integration. IEEE Journal of Photovoltaics.

[23] Woelk EG, Krautle H, Beneking H (1986). Measurement of low resistive ohmic contacts on semiconductors. IEEE Transactions on Electron Devices.

